# Spectral index selection method for remote moisture sensing under challenging illumination conditions

**DOI:** 10.1038/s41598-022-18801-9

**Published:** 2022-08-25

**Authors:** Christopher Graham, John Girkin, Cyril Bourgenot

**Affiliations:** 1 Department of Physics, Durham University, Durham, DH1 3LE UK; 2 Precision Optics Laboratory, Durham University, Sedgefield, TS21 3FB UK

**Keywords:** Climate sciences, Environmental sciences

## Abstract

Remote sensing using passive solar illumination in the Short-Wave Infrared spectrum is exposed to strong intensity variation in the spectral bands due to atmospheric changing conditions and spectral absorption. More robust spectral analysis methods, insensitive to these effects, are increasingly required to improve the accuracy of the data analysis in the field and extend the use of the system to “non ideal” illumination condition. A computational hyperspectral image analysis method (named HIAM) for deriving optimal reflectance indices for use in remote sensing of soil moisture content is detailed and demonstrated. Using histogram analysis of hyperspectral images of wet and dry soil, contrast ratios and wavelength pairings were tested to find a suitable spectral index to recover soil moisture content. Measurements of local soil samples under laboratory and field conditions have been used to demonstrate the robustness of the index to varying lighting conditions, while publicly available databases have been used to test across a selection of soil classes. In both cases, the moisture was recovered with RMS error better than 5%. As the method is independent of material type, this method has the potential to also be applied across a variety of biological and man-made samples.

## Introduction

Soil moisture content is a key metric across a variety of industries, from civil engineering and micrometeorology through to defense and agriculture^1–3^. In agriculture, accurate estimation of soil moisture is important for managing water resources and irrigation to maximise crop yield and quality^4^. The utility of remote soil moisture measurement has led to very active development in the field, with optical-near infra-red (NIR) instruments through to microwave and radio wave detectors developed for use on satellites and aircraft. Dedicated missions such as the Soil Moisture Active Passive (SMAP) satellite use L-band radiometers to recover information on soil moisture content over a global scale. While their high spatial coverage enables high temporal resolution global surveys, their low spatial resolution limits research over smaller regional areas^5,6^. A large body of research has been based on using pre-existing multispectral and hyperspectral data sources, such as the Landsat and Sentinel satellites. More recently, development of compact hyperspectral imagers has enabled the use of small unmanned aerial vehicle (UAV) systems to record high spatial resolution hyperspectral data^7,8^.

The range of instrumentation is matched by the large array of methods used to estimate soil moisture from spectral reflectance data, including spectral indices, general shape methods, and physically based radiative transfer models^9^. Each method has its own set of advantages and challenges, both in instrumentation required and in data post-processing. Spectral indices based on water absorption bands, such as Normalized Soil Moisture Index (NSMI) and Water Index SOIL (WISOIL), are simple to compute, but operate close to atmospheric absorption bands, requiring high quality atmospheric compensation post processing^10–12^.

Radiative transfer models, such as those based on the Kubelka–Monk model or the multilayer radiative transfer model of soil reflectance (MARMIT) model, can be more robust to atmospheric effects than absorption band indices^13,14^. By inverting the radiative transfer model, and fitting observed reflectance spectra to a known dry soil spectrum, using one or more parameters, an estimate of soil moisture content can be made using a variety of wavelengths. One major downside to these methods is the reliance on prior soil reflectance measurements. Soil spectral measurements are sensitive to sampling conditions such as temperature, humidity, source-sample-instrument geometry^15–17^. The spectra can also be affected by sample preparation, such as differing sieving, grinding or pulverization methods^18^. This can make close fitting of spectra acquired through remote sensing to lab calibration data difficult.

In order to work around the difficulties of comparing spectra recorded in different environments over a broad spectral range, it is possible to invert and fit these radiative transfer models using only a single wavelength band^19^. When using this method, care must be taken to fully characterise the noise of the camera sensor under measurement conditions, as small fluctuations can have a significant impact on the model inversion. Additionally the complexity of the models can result in many local minima being found during the inversion process, necessitating the use of more robust and computationally expensive optimisers compared to index methods.

The aim of this work was to develop a method for finding simple, robust spectral indices that could be used for remote sensing of soil moisture content under a variety of lighting conditions. Using an automated method to determine best contrast within a set wavelength range, indices using wavelengths less impacted by atmospheric absorption can be found. By using histogram based image analysis, with no assumptions made about the material under investigation, this method has potential to be applied to other materials and physical situations. As the focus of this work is to support the use of compact hyperspectral imagers designed for use on drones, the wavelength range analysed is restricted to the range available for compact, light weight cameras available commercially, which are InGaAs sensors.

In the following section, the Hyperspectral Image Analysis Method (HIAM) is described. In the first part of this section, we describe how the two optimal spectral indices are determined to achieve both wet and dry histogram separation and narrowness. In the second part, different contrast ratios are compared in term of wavelength determination. In the section “Experimental Data”, HIAM was then tested to recover soil moisture content under both controlled and solar illumination. In the section “Verifying with other soils”, the methods was computationally tested on publicly available databases of soil reflectances. Finally, the accuracy of the method and its range of application are discussed in the section “Discussion”.

## Image analysis method

The analysis in this section was initially based on work done by David Kim et al.^20^. The main goal of this work was to create a method for automatically searching for suitable wavelengths to use in calculating soil moisture content from soil reflectance data under changeable lighting conditions. To do this, an automated method based on histogram and contrast space analysis was devised to search through a user selectable wavelength range to find a suitable ratio providing high contrast between wet and dry soils. Ideally, a ratio would be found so that, when applied to a datacube containing both wet and dry soil, the resulting histogram would be clearly bimodal.

**Figure 1:**
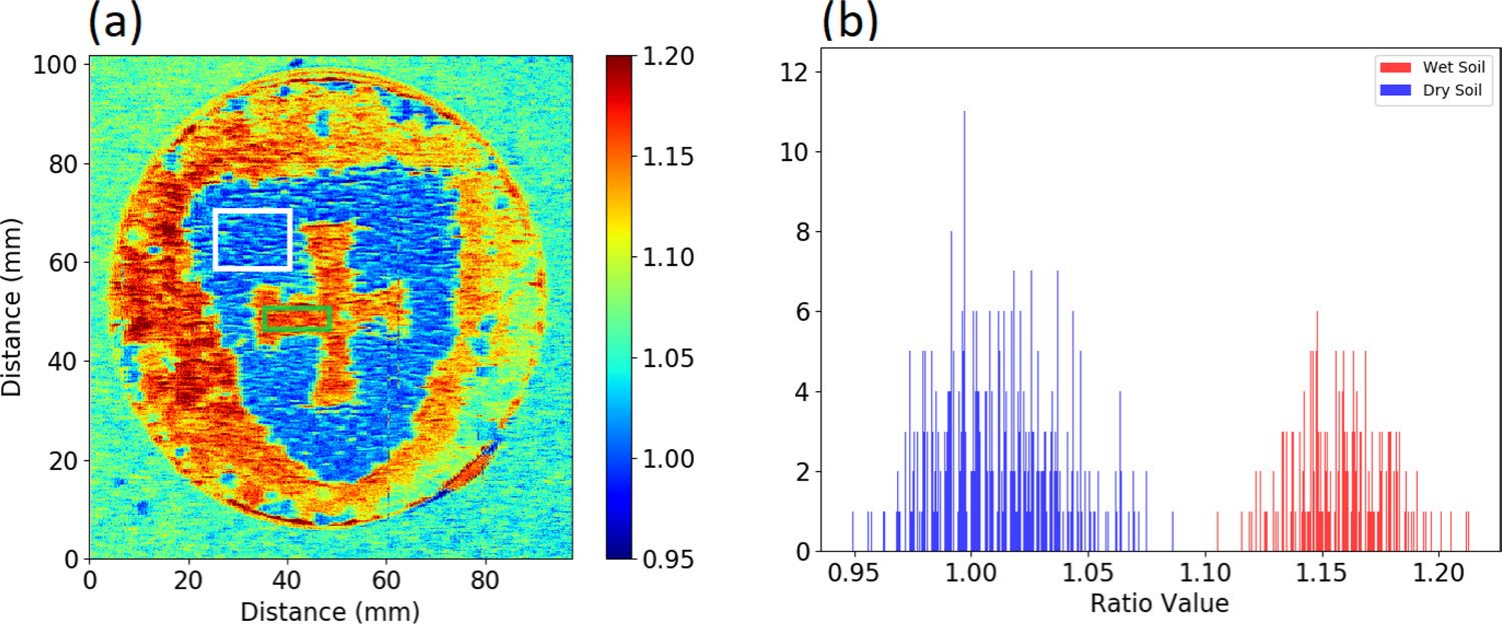
(**a**) Ratio map of a local soil, wetted in the shape of the Durham University Shield, with the central cross and exterior (red end of the colour bar) being saturated. ROIs for wet and dry soil used in the histogram are shown, marked in green and white respectively (**b**) Histogram of per pixel ratio values within the marked ROIs.

This is demonstrated in Fig. 1. Figure 1a shows the per pixel ratio of the measured reflectance at 1602/1516 nm of a local soil sample, prepared with specific wet and dry areas. A histogram comparing the per pixel ratios between the wet soil in the cross and dry soil in the shield of the Durham University logo is shown Fig. 1b, with a clear separation shown between the different moisture levels.

In order to determine the best wavelengths and ratio type (see Table 1) to produce the highest contrast between wet and dry soil, the metrics describing a useful histogram had to be identified. It was decided that the two main factors were the separation of the histogram means, and the standard deviations of the histograms. If the separation between the wet and dry histograms is too low, distinguishing between soils at different drying stages would be difficult. Similarly, ratios that produce histograms with high standard deviations could mask small changes in moisture content. This method offers a different and simpler approach to more commonly used statistical distance methods with the view to reduce the post processing required after the selection analysis. As statistical distance methods, such as the Bhattacharyya distance, could potentially output the same distance for a variety of histograms, some groups have resorted to manual sorting of results post-selection^20^.

**Table 1:** Contrast ratios considered in this analysis.

Contrast ratio	Formula
Simple	$\dfrac{Reflectance_{\lambda _1}}{Reflectance_{\lambda _2}}$
Weber	$\dfrac{Reflectance_{\lambda _1}-Reflectance_{\lambda _2}}{Reflectance_{\lambda _2}}$
Michelson	$\dfrac{Reflectance_{\lambda _1}-Reflectance_{\lambda _2}}{Reflectance_{\lambda _1}+Reflectance_{\lambda _2}}$

**Figure 2:**
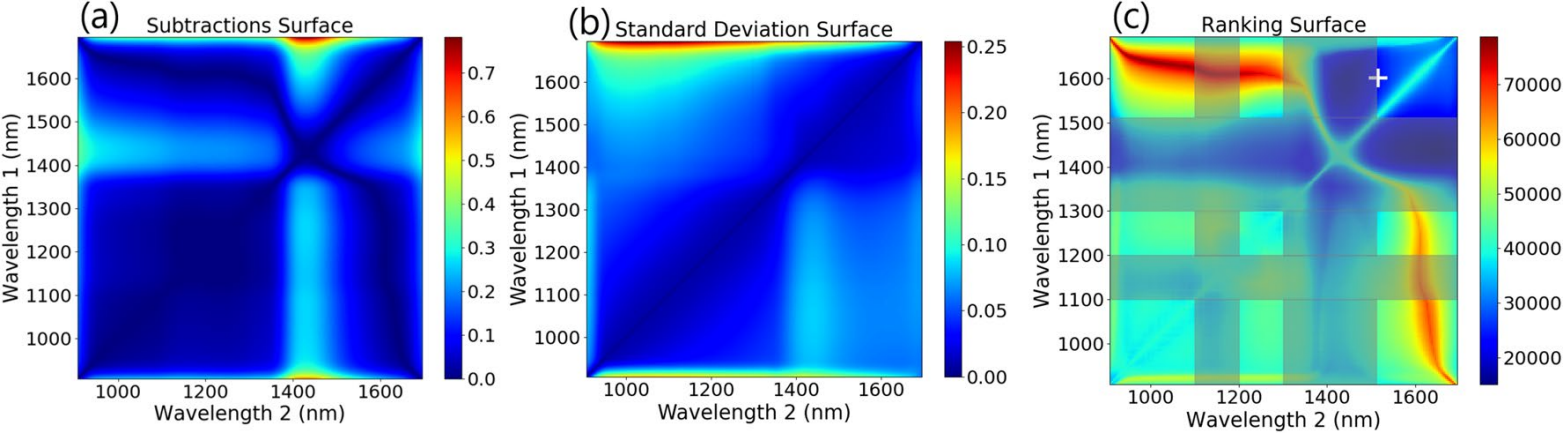
(**a**) Surface plot of the difference between reflectance ratios calculated for wet and dry soil (metric 1) (**b**) Standard deviation of per pixel reflectance ratio calculated for wet soil sample (metric 2) (**c**) Overall ranking surface, with darker blue colours best suited for differentiating wet and dry soil. The ratio picked for this analysis is marked with a white cross, chosen after atmospheric absorption bands were discarded (shown as greyed out).

To automate the process of choosing a suitable wavelength ratio, surface plots of the mean separation and standard deviation over a wavelength space were created. The mean contrast ratio separation is obtained by calculating the Euclidean distance between the mean contrast ratio of the dry soil and the mean contrast ratio of the wet soil, with the result plotted as a surface plot in Fig. 2a. For each wavelength combination covering the InGaAs sensitivity range of the FYMOS instrument used for this experiment, the following processing was performed:

1 $$\begin{aligned} metric_1 = \left| \left| \sum _{i=1}^{N_1 = ROI_{Wet}\, pixels}\frac{\left( \frac{Reflectance_{Wet}(i, \lambda _1)}{Reflectance_{Wet}(i, \lambda _2)}\right) }{N_1},\sum _{i=1}^{N_2 = ROI_{Dry}\, pixels}\frac{\left( \frac{Reflectance_{Dry}(i, \lambda _1)}{Reflectance_{Dry}(i, \lambda _2)}\right) }{N_2}\right| \right| \end{aligned}$$

To visualise how the standard deviation of the contrast ratio varies across the wavelength range, the standard deviation of the contrast ratio for each wavelength combination in the wet soil image was calculated and plotted in a similar way, shown in Fig. 2b. The following processing was performed:

2 $$\begin{aligned} metric_2 = \sqrt{\frac{\left( \sum _{i=1}^{N_1 = ROI\, pixels}\frac{\left( \frac{Reflectance_{Wet}(i, \lambda _1)}{Reflectance_{Wet}(i, \lambda _2)}\right) }{N_1} - \sum _{i=1}^{N_1 = ROI\, pixels} \frac{\left( \frac{Reflectance_{Wet}(i, \lambda _1)}{Reflectance_{Wet}(i, \lambda _2)}\right) }{N_1} \right) ^2}{N_1}} \end{aligned}$$

**Figure 3:**
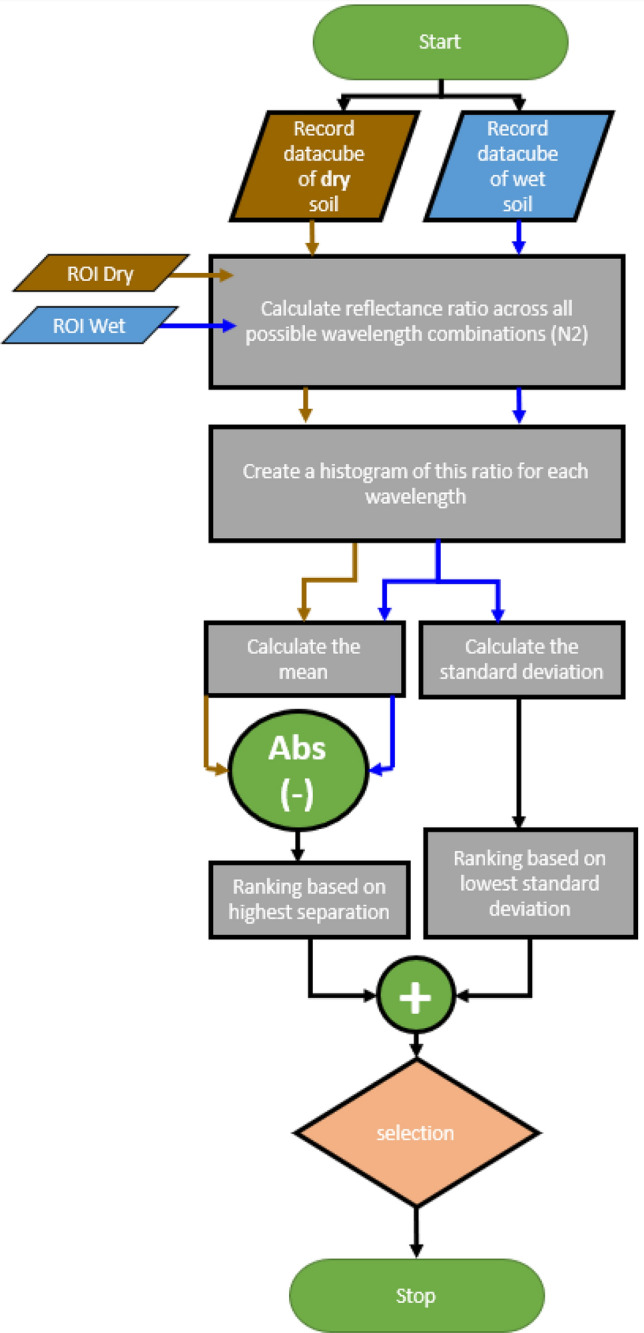
Flow chart describing the hyperspectral image analysis method (HIAM).

**Figure 4:**
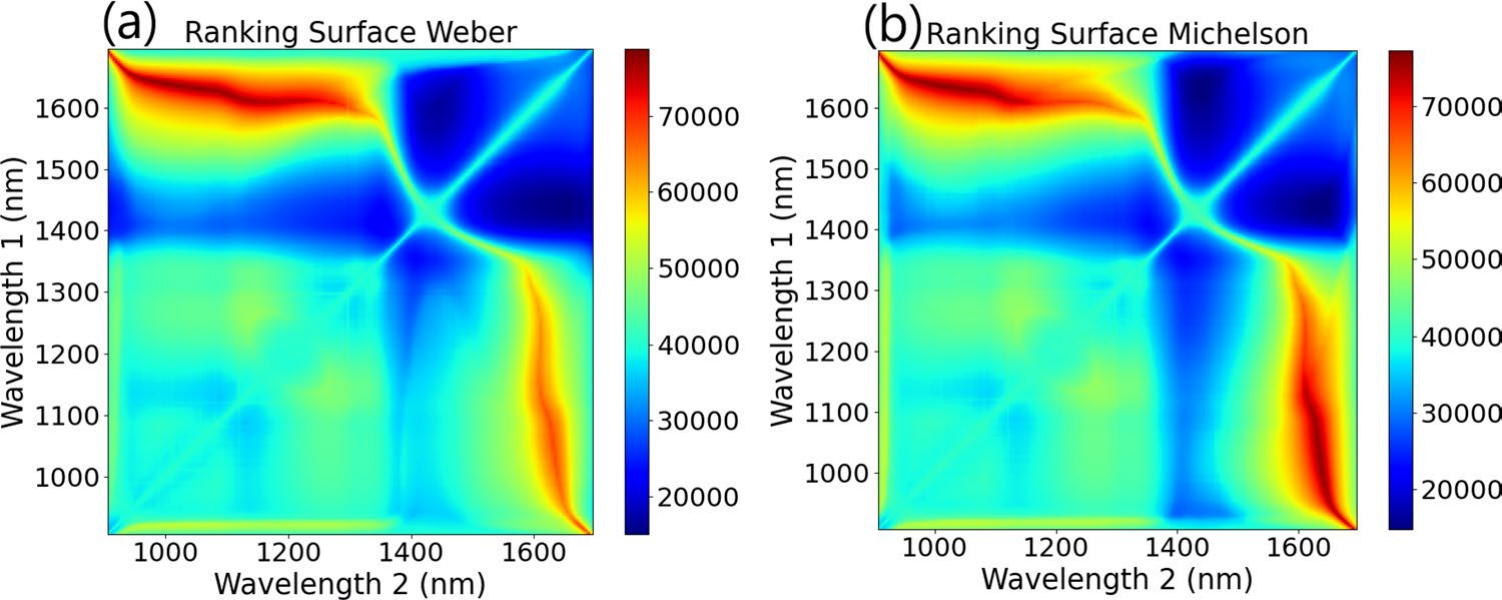
Ranking surface plots for (**a**) Weber contrast ratio and (**b**) Michelson contrast ratio.

Each wavelength pairing was then sorted and ranked based on these two surfaces, with each wavelength paring being ranked highest to lowest based on the subtraction surface, and lowest to highest based on the standard deviation surface. The rankings for each test were summed, and then plotted to form the ranking surface shown in Fig. 2c, with the idea being that the pairing with the lowest overall rank would have the best combination of high separation and low standard deviation. A flow chart outlining this process is shown in Fig. 3. The same method was applied using the simple ratio mentioned above, and using the Weber and Michelson contrast ratios, shown in Table 1. The final ranking surface for these ratios is shown in Fig. 4. All 3 contrast ratios converged towards to the same pair of wavelengths. For this application the Michelson ratio produced the lowest contrast, while the simple and Weber ratios produced similar contrast values. For simplicity, the simple ratio was chosen for further investigation.

For the soil used in this experiment, the ratio of 1524/1480 nm was found to be best. However, these wavelengths are close to the 1400 nm atmospheric water absorption band, limiting the use of the ratio under certain lighting conditions, such as under variable cloud cover. Restricting the wavelength range to wavelengths available under solar illumination, a reflectance ratio of 1602/1516 nm was chosen.

## Experimental data

To test the utility of this reflectance ratio in remote soil moisture measurement, an initial experiment was performed in the lab under controlled illumination. Soil samples were prepared by placing oven dried, sieved soil into 9 cm diameter petri dishes. The dishes were then hydrated to saturation, and oven dried at 60 °C until reaching the desired weight. The samples were then sealed and allowed to cool for 24 h to aid in uniform distribution of water content.

**Figure 5:**
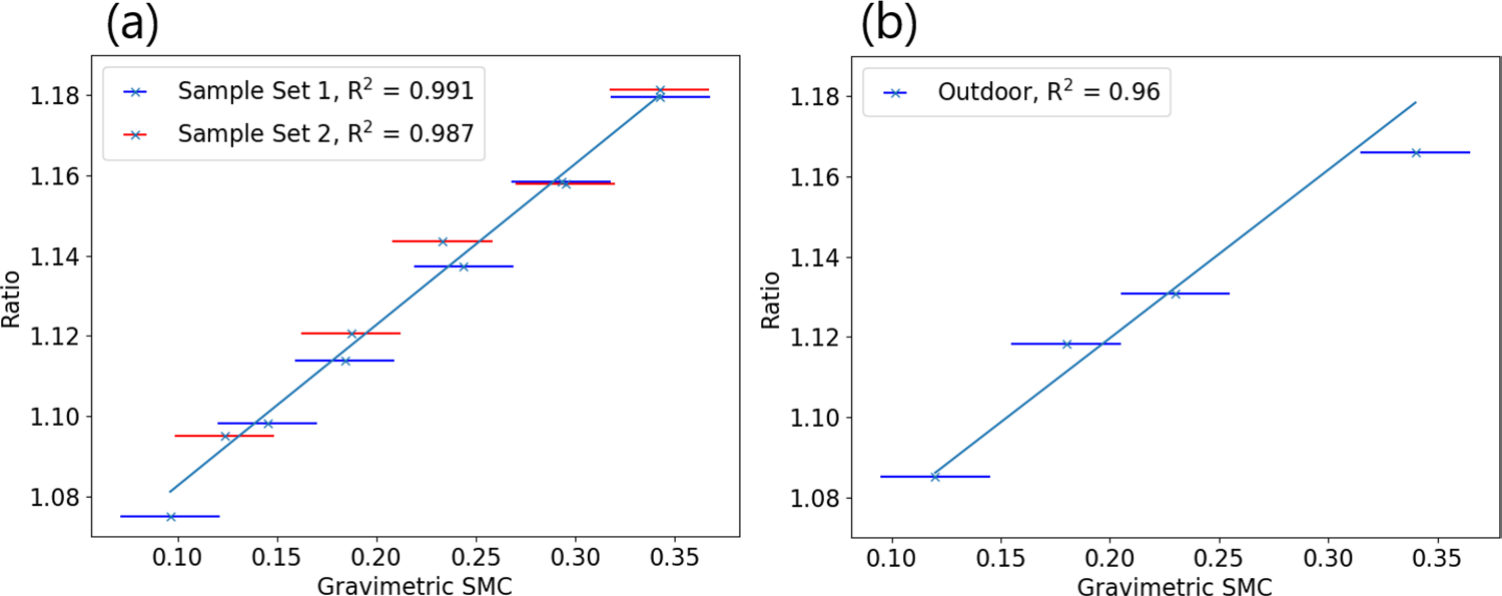
(**a**) Soil ratios from two sets of samples plotted against a mean calibration curve. These samples were measured under controlled illumination. The predicted and measured $SMC_g$ values agree within a 3% error. (**b**) Soil samples measured under solar illumination, plotted against the same calibration curve.

A hyperspectral image of each soil sample was then taken using the FYMOS hyperspectral imager, set up normal to the soil surface and scanned using a rotation stage^8^. The light source was an ASD Illuminator halogen lamp placed at a 15° angle to the soil surface. From the hyperspectral datacubes, a mean reflectance ratio for each sample was calculated to create a calibration curve. Two more sets of soil samples from the same area were then prepared in an identical way, with their ratios plotted against the calibration curve shown in Fig. 5a.

A similar set of soil samples were then measured outdoors under varying lighting conditions. The hyperspectral imager was set up on a tripod, orientated to prevent shadowing on the soil samples. One set of measurements was taken under a clear sky with a low winter sun (solar elevation angle of 22.5°), shown in Fig. 5b. A 50% reflectance Lambertian panel was used to measure the incident solar illumination, and to calibrate the measured soil data to true reflectance factors. The ratios calculated for these samples agree strongly with the measurements taken in the lab.

## Verifying with other soils

Using datasets provided by Dupiau et al.^21^, the wavelength ratio was tested against a range of various soil samples. As the spectra from these datasets have been captured using point spectrometers, the spatial imaging data required for the histogram analysis is not present. Instead, the best fit wavelengths found for the local soil were used for every database.

**Figure 6:**
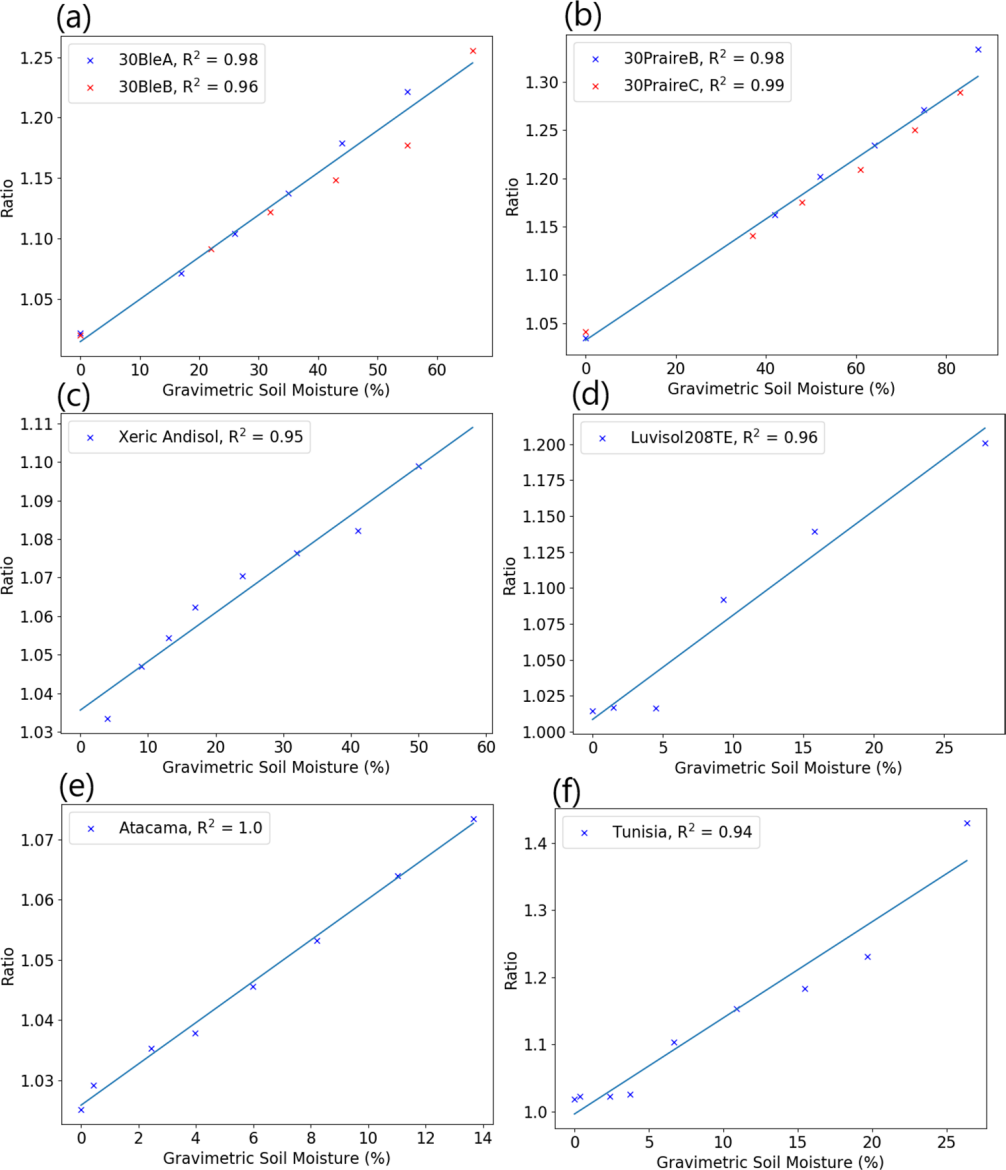
Calibration curves created for datasets provided by^21^.

The datasets chosen for validation were Les08, Lob02, Bab16 and Dup20, described in^13,21^. These datasets were chosen as their soil sample preparation methods and measurement geometry closely matched the experiments used in deriving the ratio. As each dataset contains a large number of soil samples, for brevity a set of 6 samples covering a range of soil texture characteristics was chosen for illustration here. Figure 6 shows the calculated reflectance ratio plotted against gravimetric soil moisture content for a variety of hydration levels and soil compositions. Individual soil samples have been removed where there was a suspicion of specular reflectance marked in the database. In Fig. 6c–f, a calibration curve for has been calculated for each individual data set, with the fit being good for most soil types. Where multiple sets of soil samples from the same region were available, they were plotted together, with a mean best fit curve calculated. The fit between these soil samples is still good, with examples of this are shown in Fig. 6a and b.

**Figure 7:**
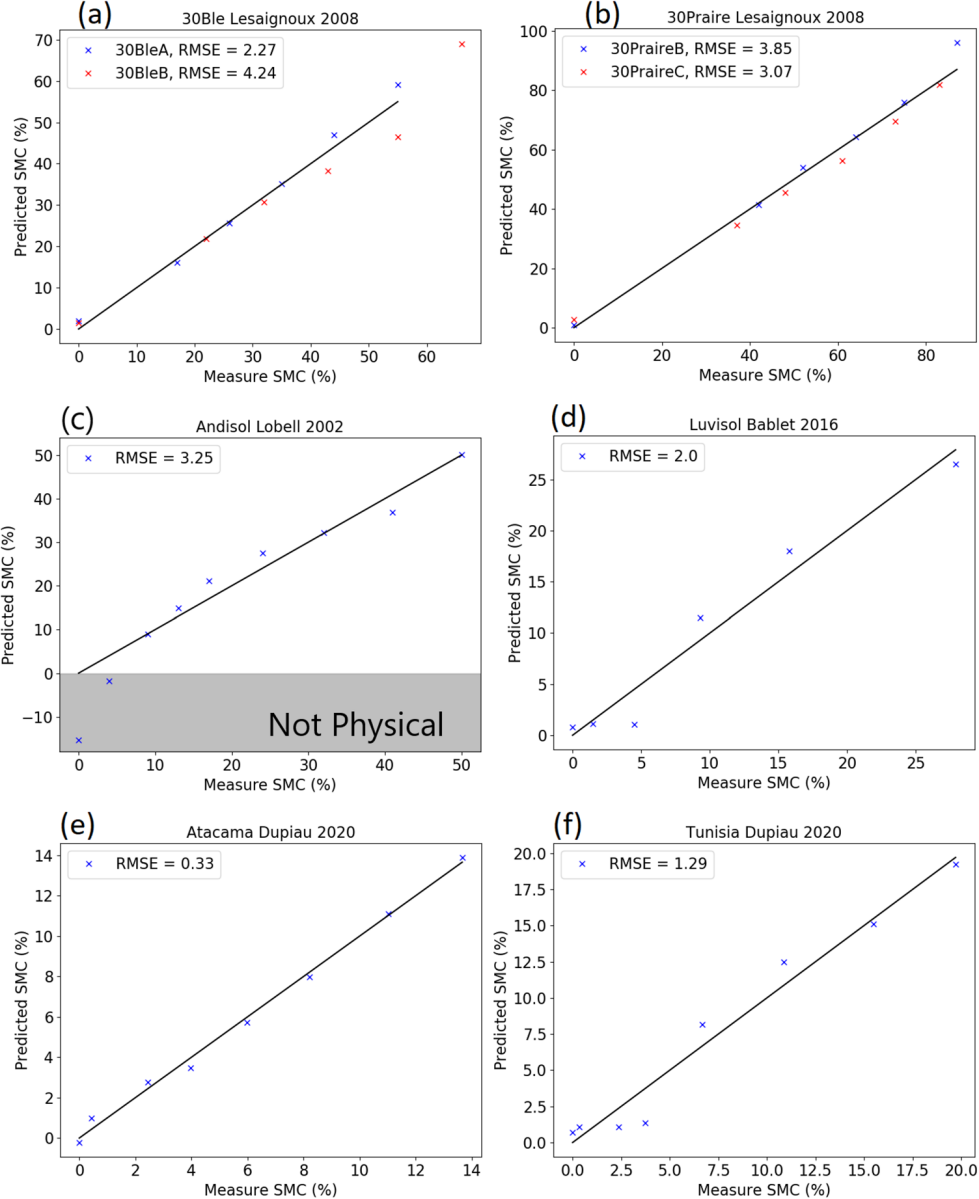
Predicted vs measured soil moisture content from calibration curves created in Fig. 6.

Feeding the calibration curves from Fig. 6 back into the same datasets, the predicted and measured soil moisture contents can be compared, shown in Fig. 7. The predicted values for Fig. 7a and b were based off the mean best fit curves for each region. Generally, there is good agreement between the predicted and measured soil moisture contents, with an RMSE under 5% for all datasets. The fit of the model tends to struggle at low soil moisture contents ($<5\%$), but tends to hold well from 10% SMC up until soil saturation. Sensitivity at lower moisture contents may be better in sandy soils, shown in Fig. 7e and f. While simple linear fits work for the majority of the soil datasets, some soils such as shown in Fig. 7c would benefit from non linear modeling. This suggests that the best performance could be found by calibrating the model independently for different soil types.

## Discussion

When performing this analysis, the two images chosen were of soil samples hydrated to the two extremes of soil moisture content, with one being oven dried and the other brought close to saturation. As this method is purely image analysis based, no underlying model of soil reflectance is included, which should make this method material agnostic. So long as the material exhibits a changing reflectance with varying moisture content, this method should be applicable. Besides soil, simple lab experiments have shown this to work with other biological samples, such as detached leaves, along with man made materials such as fabric cloth and paper.

**Figure 8:**
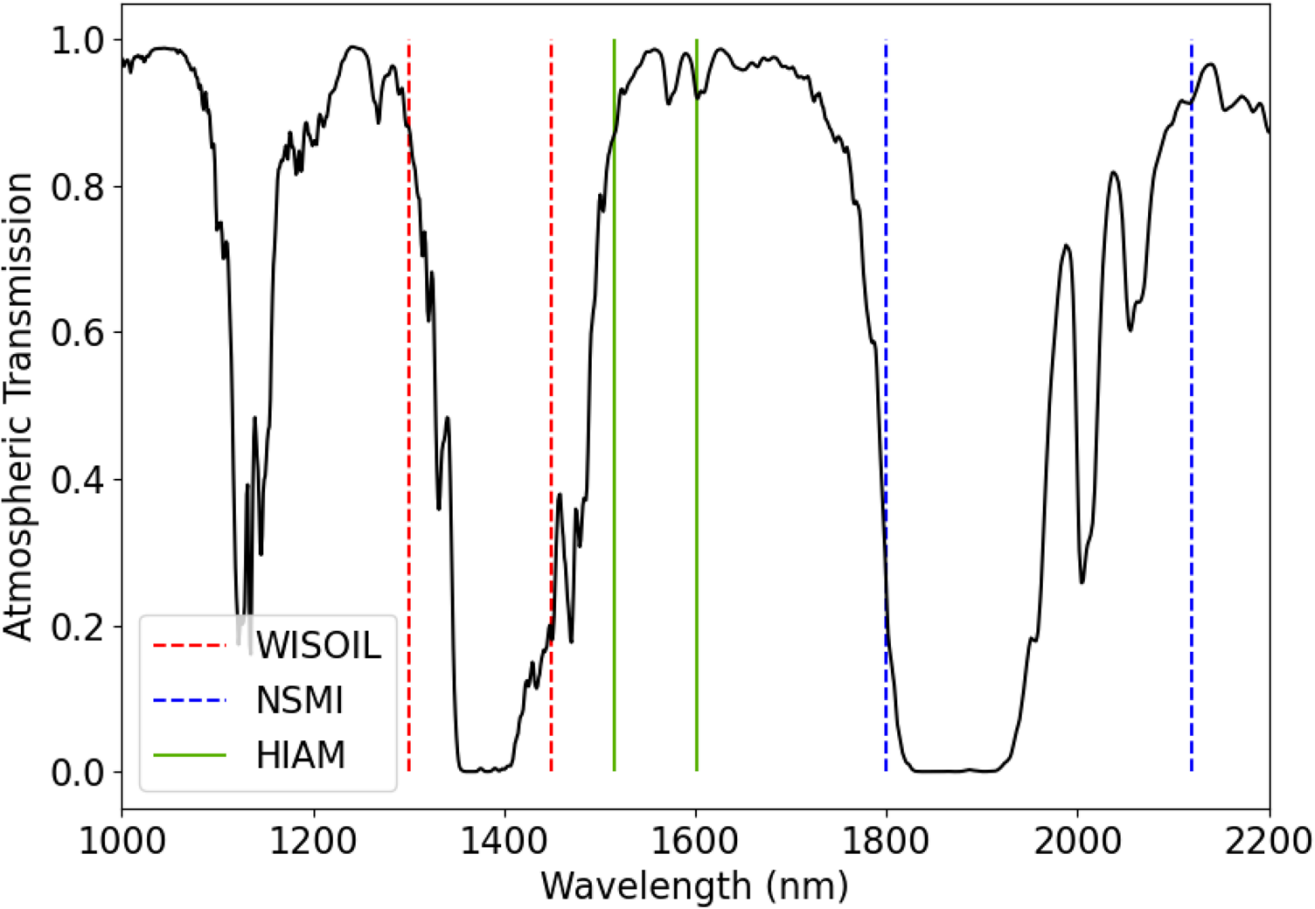
Wavelengths comprising existing indices WISOIL and NSMI, along with the index proposed in this work, imposed on atmospheric transmission spectrum computed using LOWTRAN^22^.

By carefully selecting the spectral range considered in the analysis, the index was chosen so that the wavelengths used are not heavily impacted by atmospheric absorption. This increases the robustness of the index to changing lighting conditions, as shown in Fig. 5. A comparison of the wavelengths used in this work compared to WISOIL and NSMI is shown in Fig. 8, plotted against the atmospheric transmission spectrum. While other published indices such as NINSOL and NINSON have been developed for atmospheric robustness, these use wavelengths beyond the 2200 nm cut-off found in many commercially available InGaAs focal plane arrays at present^12^.

From analysis on publicly available data sets, this index performs best at medium to high soil moisture contents. The performance is similar across a variety of soil classes, with marginally lower RMSE values found for clay loam/silty clay loam soils, where soil class data is available. However, only around half of the data sets contained textural information, making conclusions on the effects of soil texture difficult to draw.

## Conclusions

A computational image analysis method for deriving reflectance indices for remotely recovering soil moisture content has been presented. From hyperspectral imagery of local soil samples, a simple reflectance index was identified, with the wavelengths chosen to enable the use of the index easily under solar illumination. Using local soil samples and publicly available databases, the index was tested in both laboratory and field conditions, and across a variety of soil classes, with an RMSE under 5% for all data sets. With the method based entirely on hyperspectral image analysis, this method can be applied to materials beyond soil, including both biological and man made materials.

## Data Availability

The datasets generated during and/or analysed during the current study are available from the corresponding author on reasonable request.
